# Mental Health and Coping in the Shadow of the COVID-19 Pandemic: The Israeli Case

**DOI:** 10.3389/fpubh.2020.568016

**Published:** 2021-01-12

**Authors:** Mally Shechory Bitton, Avital Laufer

**Affiliations:** ^1^Department of Criminology, Ariel University, Ariel, Israel; ^2^Behavioral Science, Netanya Academic College, Netanya, Israel

**Keywords:** coronavirus, psychological distress, age, coping, resilience

## Abstract

The COVID-19 pandemic caught the entire world off guard. Israel, similar to all other nations, was forced to cope with the unknown. “Flattening the curve” of infections has become a common term among specialists and decision makers, while explaining restricting measurements taken toward the population. Israelis, who had previously learned to deal with life under constant security threat, are now facing a new reality. The purpose of the study was to check how Israelis are psychologically affected by and coping with the COVID-19 pandemic. The study included 925 Israelis divided into three groups: ages 18–29, 30–59, and 60–88. The data were collected between March 31 and April 8, 2020, when it was already clear that this is a global plague, the country's borders were closed, and the government's directive for citizens was to remain at home while imposing limitations on the public and private sectors. The current study examined psychological distress among the three age groups as well as the associations between levels of distress, resilience, and coping strategies. Levels of distress were measured via the BSI-18 that measures anxiety, depression, and somatization. Resilience was measured using the Connor-Davidson CD-RISC scale. Coping was measured by the short version of the COPE. Psychological distress was associated with being in a younger age group, being a woman, having economic concerns, use of emotion and problem focused coping, and lower resilience. The study also found that concern for the health of family members was the strongest concern among all age groups but was highest among the younger age group. It was also found that those in the younger age group suffered from higher levels of depression, anxiety, and somatization compared to the older age group. The middle age group suffered from elevated levels of anxiety and somatization compared to the older age group. Although the older age group was the most vulnerable to the coronavirus, in this study age was found to be a protective factor from psychological distress. The results of the study suggest the need to consider the younger age group as a risk group, which hence needs to be addressed as the focus of intervention programs. It appears that the concern for their loved ones takes a heavy toll on the younger generation, and this should be considered a major source of stress.

## Introduction

The 2019 Coronavirus (COVID-19) pandemic caught the whole world “off guard.” It first emerged in late December 2019 in Wuhan, China, and spread nationwide between December 2019 and early 2020 (1). On January 30, 2020, the World Health Organization (WHO) declared the COVID-19 outbreak a public health emergency of international concern. Israel was not spared. On January 30, Israel banned all flights from China, expanding this 2 weeks later to include more Asian countries. On March 12 all universities, schools, and kindergartens were closed, switching to remote study methods. On March 19, Israeli Prime Minister Benjamin Netanyahu declared a national emergency. Israelis were not allowed to leave their homes, unless absolutely necessary. Excluding essential services (food shops, pharmacies, and banks), which remained open, everything was closed. The restrictions were toughened during the days leading up to the closure, which was a prohibition on leaving one's house for a distance of more than 100 m, meetings with others who do not live in the same household, and so on. The national unemployment rate rose from 3.4 to 27% in April. Mandatory face masks outside the home was introduced on April 12, and the restrictions were gradually lifted from April 19 until the Israeli economy resumed its routine.

The current study was conducted during the peak of the closure, from March 30 to April 8. At this time there was a real concern that the pandemic would get out of hand and Prime Minister Benjamin Netanyahu, together with the Ministry of Health and its Director General appeared on television almost every evening in order to explain the severity of the pandemic and warned of a forecast of thousands of casualties and tens of thousands inflicted if the closure would not be maintained. The concern that it would not be possible to provide a medical response and that the crisis was threatening to overwhelm the Israeli healthcare system was reiterated. The rate of those diagnosed with Coronavirus rose from 4,695 cases on March 30 to 9,404 by April 8, and the number of deaths rose from 12 on March 30 to 73 on April 8.

Israeli society is used to coping with crisis situations that include war and security threats, but Israel last coped with a pandemic event of global dimensions in the 1950s, in the case of the polio pandemic.

Research on how Israelis cope with security threats indicates processes of habituation after periods of tension and anxiety (2, 3). These processes were observed both in mental and physical contexts. For example, Levav et al. (4) examined health service use among the general population in response to terrorism. They found that, with few exceptions, the residents did not seek increased help from psychiatric services during the study period. In another study, Ponizovsky et al. (5) looked at the association between psychological distress and mortality. Supporting their assumption that Israelis are conditioned to adjust to these life stresses, they found that exposure to security threats (i.e., war, combat, and terror) had no association with overall mortality or cause-specific mortality.

Nonetheless, the coping of Israeli society with a non-security threat with features of a pandemic, such as the situation formed following exposure to COVID-19, has hardly been studied [e.g., (6, 7)]. Hence, the purpose of the current study is to examine psychological distress, coping processes, and resilience of Israeli society at the height of the pandemic and of the period of social restrictions.

A review of 24 studies documenting the psychological impact of quarantine (“the separation and restriction of movement of people who have potentially been exposed to a contagious disease,” p. 912) was carried out by Brooks et al. (8). The studies were conducted across 10 countries and included people with SARS (11 studies), Ebola (five), the 2009 and 2010 H1N1 influenza pandemic (three), Middle East respiratory syndrome (two), and equine influenza (one). One of these studies related to both H1N1 and SARS. Most studies reviewed reported negative psychological effects, including symptoms of psychological stress, anxiety, insomnia, anger, irritability, emotional exhaustion, depression, and post-trauma. Stressors also included longer quarantine duration, infection fears, frustration, boredom, inadequate supplies, inadequate information, financial loss, and stigma.

Research-based evidence on the mental health effects of the current pandemic began to arrive particularly from several studies conducted in China, where the pandemic began, as stated (1, 9). The first nationwide survey of psychological distress among Chinese people in the COVID-19 epidemic was conducted by Qiu et al. (1). They found that almost 35% (*N* = 52,730) of the respondents experienced psychological distress, with significantly higher psychological distress among women and among individuals between 18 and 30 years of age or above 60. Going forward, a systematic review conducted by Xiong et al. (10) shows that although early studies from China documented higher distress among older adults, later studies from Western countries usually found lower rates of distress among older adults relative to other age groups. Similar findings were also found among Israeli older adults (7).

However, studies suggest that exposure to stressful life events retains a stable equilibrium without reactive psychopathology. A consistent body of research suggests that a majority of those who were exposed to stressful and traumatic events retain a stable equilibrium without reactive psychopathology (11, 12). The growing focus on health promotion and well-being, shifting emphasis away from pathogenic to salutogenic factors provides an opportunity to examine the role of resilience and the coping strategies in health.

Different operational definitions and corresponding methodology for measuring resilience have been offered (13). Connor and Davidson (14) define resilience as a personality trait that embodies the personal qualities that enable one to thrive in the face of adversity. In other words, it is a set of protective factors (e.g., close relationships with family and community, optimistic outlook, embracing challenges) allows an individual to have a positive response to adverse events (14). Reference to resilience as a personality trait is expressed in the questionnaire authored by them, named *The Connor–Davidson Resilience Scale* (14) that is used in this study, as well as in others [e.g., (15)]. Research findings note that trait resilience is a relatively stable personality feature (16), that was found to be associated with lower levels of distress (e.g., depression, anxiety, sleep disorders, and PTSD) and better physical and mental health (12, 14, 16, 17).

Resilience has been associated with coping strategies, in the context of adverse events (18). In the current study, we use the notion proposed by Lazarus and Folkman (19, 20) that coping strategies are cognitive and behavioral efforts to manage specific external and/or internal demands appraised as taxing or exceeding the resources of the person dealing with stressful situations and events. Coping strategies may yield either positive or negative results. They were found to be an important feature, which moderates the association between exposure to stress and mental health in various contexts (21, 22). Lazarus and Folkman (19) suggested two major forms of coping: problem-focused (dealing with stress sources and taking proactive steps to change them) or emotion-focused (serving to reduce the emotional stress resulting from such situations) [See also: (23)].

Use of problem-focused strategies usually shows more negative correlations with distress, and indicates good mental health (24, 25) and higher levels of resilience (22, 26). In contrast, greater use of emotion-focused coping is highly correlated with high levels of psychological distress [e.g., (24, 27–29)].

However, the distribution of coping strategies is not so dichotomous (30). Several studies have shown that both coping strategies were positively correlated with pathogenic (e.g., PTS symptoms) as well as with salutogenic factors (e.g., resilience, post traumatic growth) (3, 22). It was also found that emotion-focused strategies may also be beneficial in situations perceived as uncontrollable or in the absence of a viable solution (e.g., terrorism exposure and security threats) (31–34). In these cases, it even might be better to use emotion-focused coping, since this strategy may reduce the negative psychological effects of the scenario/event without confronting it directly (30).

The Coronavirus revealed different risk levels for different age groups, with a higher risk for people aged 60 or older. Studies on nation-wide populations indicate that age is a major factor in addressing mental health outcomes (35). Therefore, in the current study we aim to examine the levels of psychological distress and concern about health and financial situation during this special period among different age groups, as well as the relations to resilience and coping strategies across ages and for specific age groups. Three age groups were examined: older adults (60+), middle group (59–30), and younger (22–33). The age groups were selected based on former studies that utilized similar age group examinations of mental health outcomes (1, 35).

We hypothesized that participants would report high concerns for their own and their families' health regarding the COVID-19 pandemic, as well as high concerns for the financial implications of the COVID-19 pandemic. Furthermore, we hypothesized that a higher level of health concerns and psychological distress would be reported by the older adults age group compared with the other age groups, since this age group was at the highest risk of dying after contracting the virus. Finally, negative correlations were hypothesized between the level of psychological distress, and resilience and problem-focused coping, and a positive correlation between level of distress and emotion-focused coping.

## Method

### Participants

Nine hundred and twenty-five participants took part in the study. They were divided into three groups: younger, aged 18–29, *N* = 189 (20.4%); middle, aged 30–59, *N* = 473 (51.1%), and older adults, aged 60–88, *N* = 263 (28.4). Most of the respondents in the middle aged and older adults groups had children (90.3 and 97%, respectively). Only 13.8% of the younger group had children. Among those with children, they had up to 11 children, with a greater number of children in the older adults group (*M* = 3.05, SD = 1.18) than in the middle aged group (*M* = 2.73, SD = 1.42), with the younger group having the fewest children (*M* = 0.35, SD = 0.082) (*F*_(2,854)_ = 223.70, η^2^ = 0.344, *p* < 0.001).

As seen in Table 1, there were 71% females, with no meaningful gender differences by age group. Most participants in the middle age and older adults groups were married or in a steady relationship (about 80%), while most participants in the younger group were single (61%), a significant difference. Most respondents had an academic education, yet to a higher extent in the middle aged group (75%) than in the younger (60%) or older groups (57%).

**Table 1:** Distribution of background variables by age group (*N* = 925).

		**Total sample**	**Young**	**Middle**	**Older adults**	**Difference**
		***N* (%)**	***N* (%)**	***N* (%)**	***N* (%)**	
Gender	Male	268 (29.0)	60 (31.7)	118 (24.9)	90 (34.2)	χ^2^(2) = 7.95 (*p* = 0.019)
	Female	657 (71.0)	129 (68.3)	355 (75.1)	173 (65.8)	
Family status	Married, in a relationship	656 (71.0)	70 (37.2)	373 (78.9)	213 (81.0)	χ^2^(2) = 131.02 (*p* < 0.001)
	Other (single, divorce, widower)	268 (29.0)	118 (62.8)	100 (21.1)	50 (19.0)	
Education	Secondary	194 (21.0)	63 (33.3)	66 (14.0)	65 (24.8)	χ^2^(4) = 48.95 (*p* < 0.001)
	Vocational	114 (12.4)	13 (6.9)	53 (11.2)	48 (18.3)	
	Academic	615 (66.6)	113 (59.8)	353 (74.8)	149 (56.9)	

*The Bonferroni correction for multiple comparisons yields p = 0.017*.

### Measurements

#### Personal Data

Data were gathered regarding gender, age, religiosity, level of education, number of children, age of youngest child, type of residential town, residential region.

#### Coronavirus Objective and Subjective Exposure

This measure was devised for the current study. Questions were asked regarding the current time—during closure due to COVID-19. Did you contract the Coronaivrus? Were you admited to a hospital or quarantined at home becuase you were ill with the virus? Did someone from your family contract the Coronavirus? Did you continue working during the closure? Who is currently at home with you?

Three additional questions related to respondents' degree of concern during this period. Participants were asked to rate on a 5-point scale (1 = not at all to 5 = very much): How concerned are they that their health will be affected due to contracting the Coronavirus? How concerned are they that their family members will be affected by contracting the Coronavirus? And how concerned are they about their financial situation due to the Coronavirus crisis?

#### Coping Strategies: Measured by the COPE Scale (36)

The scale assesses two major coping strategies: problem-focused (15 items) and emotion-focused (15 items). The scale has been used extensively in Hebrew [e.g., (27, 37)]. Participants were asked to rate the extent to which they used each coping option to deal with the stressful situations caused by the COVID-19 pandemic, on a 4-point scale (0 = not at all; 3 = a great deal) (data were transformed into a 1–4 scale). In the current study, internal consistency was 0.78 for problem-focused and 0.73 for emotion-focused coping.

#### Resilience

This measure was examined by the Connor-Davidson Resilience Scale (CD-RISC; 10), which consists of 25 statements (e.g., *able to adapt when changes occur; have close and secure relationships; belief one can deal with whatever comes and having control of one's life*). Each statement is rated by respondents in terms of the extent of their agreement with it over the previous month (0 = not at all to 4 = true nearly all the time). This scale has been used among the Israeli population and has shown good predictive validity and internal consistency (15, 22). Total CD-RISC scores representative of resilience were utilized for this study (α = 0.89).

#### Psychological Symptoms

Psychological symptoms were assessed using the BSI-18 (38), which is a self-report symptom checklist measure consisting of 18 items taken from the 53-item Brief Symptom Inventory [BSI; (39)]. Each BSI-18 item describes a symptom to be rated by respondents on a five-point scale according to how much they were bothered by the symptom in the previous week. Scores on the 18 items are summarized on the Global Severity Index (GSI) (α = 0.92) and regarding three symptom scales: Somatization (α = 0.82), Depression (α = 0.82), and Anxiety (α = 0.86), each comprising six items.

### Procedure

For collecting the data, we used a cross-sectional anonymous online questionnaire. The data was collected between March 31 and April 8, 2020, a time when the Israeli government had issued a directive for citizens to isolate themselves at home and minimize face-to-face interaction. Thus, potential respondents were electronically invited by existing research respondents. The participants completed the questionnaires through an online survey platform. Then the raw data was transferred into a database. The online questionnaire offered the necessary assurances of anonymity to allow respondents to give accurate data surrounding sensitive issues, which is particularly relevant in the field of mental health. All respondents provided informed consent. The study was approved by the ethical standards of the Institutional Review Board (IRB) of the University.

### Data Analysis

Data were analyzed with SPSS v. 26. Internal consistencies were calculated for the research variables, and the research variables were computed with item means or sums. As BSI scores were positively skewed they were log transformed. Background characteristics of the respondents were described with means and standard deviations for continuous variables and frequencies and percentages for categorical variables. The three age groups were compared through analyses of variance and chi-squares, respectively. Means, standard deviations, and intercorrelations for the research variables were described. Analyses of variance with *post-hoc* estimated marginal means with the Bonferroni correction for multiple comparisons were calculated to compare the research variables across the three age groups. A multiple hierarchical regression for the total BSI score with the research variables was calculated. The first step included gender (1-males, 0-females) and the age groups (entered as two dichotomous variables: younger vs. others, and older adults vs. others). The second step included the variables of concerns, coping strategies, and the total score for resiliency. The Bonferroni correction for multiple comparisons was applied in all tables.

## Results

### Coronavirus Objective and Subjective Exposure

Only one participant had contracted the virus and remained at home. Only 10 family members of the respondents (1.1%) had contracted COVID-19. Table 2 presents the differences between the groups in objective and subjective exposure.

**Table 2:** Distribution of objective and subjective exposure by age group (*N* = 925).

		**Total sample**	**Young**	**Middle**	**Older adults**	**Difference**
		***N* (%)**	***N* (%)**	***N* (%)**	***N* (%)**	
Lives with (at present)	Alone	91 (9.9)	20 (10.6)	33 (7.0)	38 (14.6)	χ^2^(2) = 10.83 (*p* = 0.004)
	Family and mate	828 (90.1)	168 (89.4)	437 (93.0)	223 (85.4)	
Employment during Coronavirus	Yes- works	464 (56.9)	94 (50.8)	299 (64.9)	71 (41.8)	χ^2^(2) = 3.58 (*p* < 0.001)
	No- stopped	352 (43.1)	91 (49.2)	162 (35.1)	99 (58.2)	
	**Range**	***M*** **(*****SD*****)**	***M*** **(*****SD*****)**	***M*** **(*****SD*****)**	***M*** **(*****SD*****)**	
Concerned about own health	1–5	2.70 (1.16)	2.59 (1.15)	2.69 (1.17)	2.81 (1.14)	*F*_(2,919)_ = 1.98
						(*p* = 0.139), (η^2^ = 0.004)
Concerned about family health	1–5	3.56 (1.14)	4.08 (0.98)	3.49 (1.15)	3.32 (1.12)	*F*_(2,920)_ = 28.32 (*p* < 0.001), (η^2^ =0.058) 1 > 2, 3
Concerned about financial status	1-5	2.73 (1.24)	2.84 (1.33)	2.78 (1.21)	2.58 (1.20)	*F*_(2,919)_ = 2.68 (*p* = 0.069), (η^2^ = 0.006)

*The Bonferroni correction for multiple comparisons yields p = 0.010*.

Participants in the three groups were mainly living with family or with their spouse during the COVID-19 epidemic. Of those who had been employed, about 43% had to stop working. Their rate was highest in the older adults group (58%), lower in the younger group (49%), and lowest in the middle aged group (35%). Most respondents had an academic education, yet to a higher extent in the middle aged group (75%) than in younger (60%) and older groups (57%).

Examination of levels of concern showed that participants were moderately concerned about their own health (*M* = 2.70) with no significant group difference. They were more concerned about their family's health (*M* = 3.56) than about their own health (*t*_(923)_ = 26.62, *p* < 0.001). Concern for the family's health was highest among the younger participants (*M* = 4.08), and lower among both middle aged (*M* = 3.49) and older adult (*M* = 3.32) participants. Further, the participants were moderately concerned about their financial state (*M* = 2.73), with no significant age group differences. Concern about family members' health was the highest, compared to both concerns about own health and about participants' financial status (*F*_(2,1840)_ = 295.40, *p* < 0.001, η^2^ = 0.243). Further, the interaction between the type of concern and age group was significant (*F*_(4,1840)_ = 19.81, *p* < 0.001, η^2^ = 0.041). Interpretation revealed that for the younger group concern about the family's health was highest, then financial concerns (*p* < 0.001), and lowest was the concern about their own health (*p* = 0.034). In the middle aged group, concern about the family's health was higher than both financial concerns and the concern for their own health (*p* < 0.001). In the older adults group, concern about the family's health was highest, followed by concerns about their own health (*p* < 0.001), and lowest was financial concerns (*p* < 0.001).

### Intercorrelations for the Research Variables

Table 3 presents means, standard deviations, and intercorrelations for the research variables. Problem focused coping was moderate low, and emotion focused coping was lower (*t*_(923)_ = 21.38, *p* < 0.001). Resiliency was moderate high, and all BSI mean scores were low.

**Table 3:** Means, standard deviations, and intercorrelations for the research variables (*N* = 925).

	***M* (*SD*)**	**2**.	**3**.	**4**.	**5**.	**6**.	**7**.
1. Coping: problem (0–3)	1.35 (0.55)	0.51^***^	0.16^***^	0.27^***^	0.16^***^	0.33^***^	0.18^***^
2. Coping: emotion (0–3)	1.01 (0.36)		−0.01	0.43^***^	0.30^***^	0.44^***^	0.35^***^
3. Resiliency: total (0–100)	67.39 (13.32)			−0.34^***^	−0.24^***^	−0.29^***^	−0.36^***^
4. BSI- total score (0–4)	0.54 (0.51)				0.80^***^	0.93^***^	0.88^***^
5. BSI- somatization (0–4)	0.29 (0.47)					0.66^***^	0.55^***^
6. BSI- anxiety (0–4)	0.78 (0.68)						0.71^***^
7. BSI- depression (0–4)	0.57 (0.59)						

*** *p < 0.001*.

*The Bonferroni correction for multiple comparisons yields p = 0.002*.

Significant correlations were found among the research variables. Problem focused coping was positively related with emotion focused coping, resiliency, and all BSI scores. Emotion focused coping was positively related with all BSI scores as well. Resiliency was negatively related with the BSI scores.

Table 4 presents group differences in the research variables, controlling for gender (1-male, 0-female) and education level (1-academic, 0-less than academic). Family status was not controlled for, as it had too low a variance in two of the three groups and was thus group specific.

**Table 4:** Distribution of the research variables by age group (*N* = 925).

		**Total sample**	**Young**	**Middle**	**Older adults**	**Difference**	
	**Range**	***M*** **(*SD*)**	***M*** **(*SD*)**	***M*** **(*SD*)**	***M*** **(*SD*)**		
Coping- problem focused	0–3	1.35 (0.55)	1.34 (0.53)	1.43 (0.54)	1.22 (0.54)	*F*_(2,919)_ = 7.07 (*p* < 0.001) (η^2^ = 0.025)	2 > 3
Coping- emotion focused	0–3	1.01 (0.36)	1.01 (0.32)	1.03 (0.38)	0.99 (0.35)	*F*_(2,919)_ = 0.63 (*p* = 0.534) (η^2^ = 0.001)	—
Resiliency- total score	0–100	67.39 (13.32)	67.68 (12.52)	67.70 (13.48)	66.61 (13.6)	*F*_(2,915)_ = 0.48 (*p* = 0.618) (η^2^ = 0.001)	—
BSI- total score	0-4	0.54 (0.51)	0.73 (0.63)	0.54 (0.50)	0.42 (0.39)	*F* (2, 908) = 20.80 (*p* < 0.001) (η^2^ = 0.044)	1 > 2 > 3
BSI- somatization	0–4	0.29 (0.47)	0.40 (0.57)	0.29 (0.46)	0.20 (0.38)	*F*_(2,908)_ = 10.36 (*p* < 0.001) (η^2^ = 0.022)	1 > 2 > 3
BSI- anxiety	0–4	0.78 (0.68)	0.95 (0.78)	0.81 (0.70)	0.60 (0.52)	*F*_(2,908)_ = 14.36 (*p* < 0.001) (η^2^ = 0.031)	1 > 2 > 3
BSI- depression	0–4	0.57 (0.59)	0.84 (0.76)	0.52 (0.53)	0.45 (0.49)	*F*_(2,908)_ = 24.62 (*p* < 0.001) (η^2^ = 0.051)	1 > 2, 3

*The Bonferroni correction for multiple comparisons yields p = 0.007*.

Problem focused coping was generally moderate low and was higher among the middle aged group than among the older adults group. Emotion focused coping was generally low and did not differ by group. Problem focused coping was generally higher than emotion focused coping (*F*_(1,921)_ = 364.40, *p* < 0.001, η^2^ = 0.283). Further, the interaction between coping style and age group was significant (*F*_(2,921)_ = 9.41, *p* < 0.001, η^2^ = 0.020). Interpretation revealed that in all age groups problem focused coping was higher than emotion focused coping, yet to a greater extent in the middle aged group (η^2^ = 0.264), than in the younger (η^2^ = 0.088) and older (η^2^ = 0.067) groups.

Resiliency was moderate-high and did not differ by age group. BSI mean scores were low. The total score, somatization, and anxiety were highest among the younger group, lower among the middle aged group, and lowest among the older adults group. Depression was higher among the younger group than among the middle aged and older adults groups.

### Regression

In order to assess the relationship between distress (total BSI score) and the research variables, we conducted a multiple hierarchical regression. Table 5 presents a multiple hierarchical regression for the total BSI score. The first step included gender (1-males, 0-females) and the age groups (entered as two dichotomous variables: younger group vs. others, and older adults group vs. others) (education level was not entered as it was unrelated to distress- *r* = −0.01, *p* = 0.796). The second step included the research variables of concerns, coping strategies, and the total score for resiliency.

**Table 5:** Multiple hierarchical regression for the total BSI score (*N* = 925).

	***B***	***SE***	**β**	***p***
**Step 1**				
Gender	−0.14	0.02	−0.22	<0.001
Age group- younger	0.11	0.02	0.15	<0.001
Age group- older adults	−0.06	0.02	−0.09	0.007
Adj. *R*^2^	0.09			
**Step 2**				
Gender	−0.05	0.02	−0.09	0.002
Age group- younger	0.11	0.02	0.15	<0.001
Age group- older adults	−0.04	0.02	−0.07	0.014
Concern about own health	0.02	0.01	0.06	0.086
Concern about family's health	0.01	0.01	0.05	0.171
Concern about financial status	0.03	0.01	0.12	<0.001
Coping- problem focused	0.05	0.02	0.10	0.002
Coping- emotion focused	0.24	0.02	0.31	<0.001
Resiliency- total score	−0.01	0.01	−0.33	<0.001
Adj. *R*^2^	0.38			

*F _(9,915)_ = 64.02, p < 0.001*.

*The Bonferroni correction for multiple comparisons yields p = 0.005*.

The regression model was found to be significant, with 38% of the variance explained by the total BSI score. Gender and age group were found significant, showing higher levels of psychological distress among women than men, and among the younger age group than the other groups. Higher levels of concern about the financial status was related to higher psychological distress. Greater use of emotion focused coping, as well as greater use of problem focused coping, were related to higher psychological distress. Finally, lower levels of resiliency were related to higher psychological distress as well.

## Discussion

The study was conducted at the peak of the lockdown, at a time when there was a real concern that the pandemic would get out of hand. The COVID-19 crisis had received unprecedented levels of documentation and publicity around the world. For weeks, almost every media source (newspapers, television, radio, internet) had back-to-back coverage of the coronavirus pandemic, reporting the numbers of those infected and dead and presenting frightening statistics about the hundreds and thousands of people who had died daily. Our aims were to examine psychological distress and concerns about health and about the financial implications during this unique period among different age groups. Another aim was to explore the relationship between resilience and coping on one hand and psychological distress on the other.

The research findings indicate differences between and within the groups with regard to the three types of concern explored: concern of contracting the virus, concern that a family member would contract the virus, and concern of the financial implications. Most of the respondents expressed concern particularly with regard to their family and less regarding the financial situation. The former concern was particularly high (4.08 on a scale of 1 to 5) among the younger group, consisting of those under 30. Although this was the main concern, the regression findings indicate that it was the financial concern that was found to be associated with the respondents' level of distress.

The fact that financial concerns and not concern for the family's health was associated with psychological distress can be explained by the Israeli reality at the time the study was conducted. Most of the participants in the three groups were living with family or with their spouse at the time of this study. Israeli citizens were asked to remain at home and were in fact in a state of lockdown that protected them from contracting the virus. Indeed, as evident from the participants' reports, only one participant had contracted the virus and remained at home and only 10 family members of the participants (1.1%) had contracted COVID-19. The most significant effect of the coronavirus was the need to stop working and to remain at home during the lockdown, with no knowledge of when and even whether they would return to their jobs. Most of the government efforts were directed at preventing the pandemic from spreading and, at least in the first stage when the study was conducted, less government attention was given to the financial implications.

In our estimate the high unemployment rates following the crisis, side by side with the focus on health-related means of protection, explain how considering the lockdown and the government focus on obtaining hospital equipment the citizens felt relatively protected from a health perspective and that the government was making efforts to protect them from contracting and dying from the virus. In contrast, it was the lockdown and cessation of work, as well as the lack of government clarifications regarding the financial steps that would be taken, that led to increasing concern of the financial implications. The post-study Israeli reality, evident at the time these lines are being written (mid-May 2020), reinforces this assumption. Despite the easing of the lockdown and the approval given to return to work, many citizens have lost their jobs and the unemployment rate is high, indicating distress that is currently manifested in protests and demonstrations against the government, in a request for massive financial assistance for those whose source of subsistence was affected by the pandemic. The association found between psychological distress and financial concerns certainly appears to indicate real distress. A similar association was found by Qiu et al. (1) who explained the high psychological distress found among Chinese citizens by concerns about delays in work time and subsequent deprivation of their anticipated income, possibly explaining the high stress level.

In contrast to the hypothesis whereby adults over 60 would report higher psychological distress than others, the current findings show that they displayed the lowest levels of distress while the younger group displayed the highest levels. Consistent with these results, previous studies have found lower reactivity to stress in older adults due to the COVID-19 pandemic [e.g., (7, 40)].

We assumed that the information publicized whereby the older adults group have the highest risk and the younger group, even if contracting the virus, are not at risk of death, as well as the reports of the very high death rates among older adults, would lead to higher distress among the older adults group compared to the younger group. Similar assumptions led to different findings in a study held in China also during the peak of the lockdown (1), where high levels of distress were found among both the older adult and younger groups. The Chinese researchers explained the high level of distress among the older adults group as due to the fact that the highest mortality rate during the epidemic occurred among older adults, adding that psychological distress levels were also influenced by the availability of local medical resources, the efficiency of the regional public health system, and prevention and control measures taken against the epidemic situation.

These explanations do not seem to have been compatible with the Israeli circumstances during the crisis. While particularly high death rates were reported in China, in Israel the death rate was very low, as was the rate of those infected. In Israel, the health system dealt with the cases discovered very successfully and managed to prevent an outbreak of the pandemic.

Another possible explanation is related to the attention and high level of care directed at older adults, both by the media and various aid organizations and by their families. Caring for older adults was emphasized in all possible media, side by side with warnings and instructions to protect mainly older adults who constitute a risk group, where the sentence “Protect grandpa and grandma—Keep a distance” became a popular motto. The media was flooded with photographic evidence showing that despite the physical isolation and the prohibition of contact between older adults and their family members, strong daily contact was maintained between them by digital means (Zoom, WhatsApp, etc.). The considerable social support provided to this age group might have moderated their sense of distress and loneliness. The association between social and family support has been found to be a moderator of distress and a factor that helps cope with crisis situations (22, 41). Family and personal resources seem to be relevant for explaining loneliness and psychological well-being during a critical stressful period (40). Therefore, it may be that although the oldest age group had the highest risk to their physical health, they were more capable of dealing with the psychological distress that accompanied the coronavirus.

Returning to the younger group, as mentioned above younger participants were found to have the highest levels of psychological distress compared to the other age groups. Similar findings regarding young participants were found among the Chinese during the pandemic (1). The researchers explain this finding by the fact that this age group is highly interested in the media and therefore obtain more information that may result in their higher susceptibility. Other explanations may be related to the respondents' age and not necessarily to exposure to the media, as the latter was true of all ages. The higher levels of distress and concern among this age group may be due to their younger age, which meant that they had limited previous exposure to new stressors. Thus, while in the older adults age group their long life experience granted them the ability to manage new stressors, this was not so among the younger group. Support for this assumption came from other findings showing that mental health disorders are more frequent and apparent in younger age groups, and unlike physical disorders they tend to decrease as the individual matures (35). For example, in a study conducted in Singapore following the acute respiratory syndrome (SARS) epidemic (42), greater anxiety was associated with younger age. The researchers' assumption was that this is related to differences in coping styles among younger individuals.

Examination of the differences in resilience between the age groups revealed no difference in the use of resilience and that resilience was moderate high and negatively associated with psychological distress. The fact that resilience had similar distribution across age group is in line with Connor and Davidson (14) perception of resilience as a personality trait and not the result of confronting previous life experiences. The negative association between resilience and psychological distress is in line with studies indicating that resilience protects individuals from the deleterious effects of exposure to stress and trauma (13, 16).

With respect to the findings regarding coping strategies, we found more use of problem-focused coping than of emotion-focused coping, unrelated to age. This finding is indicative of a healthy coping style. The greater use of problem-focused coping in the middle age group specifically is also understandable. This age group has a greater need to cope with the reality of being at home with young children, compared to the younger group (most of whom have no children) and the older adults group.

The positive association between use of problem-focused coping and resilience supports these explanations. Resilient individuals have been found to employ greater amounts of active coping such as problem-focused coping (26). While resilience allows an individual to respond positively to adverse events (14), coping strategies may yield either positive or negative results. As hypothesized and in line with the literature, greater use of emotion-focused coping was related to higher psychological distress (24, 28). However, the positive association between problem focused coping and psychological distress needs to be addressed. This finding contradicts research findings that indicate a reverse association between distress and problem focused coping (24, 25). However, several studies have shown that both coping strategies were positively correlated with pathogenic (e.g., PTS symptoms) as well as with salutogenic factors (e.g., resilience, post traumatic growth) (3, 22).

It was also found that emotion-focused strategies may be beneficial in situations perceived as uncontrollable or in the absence of a viable solution (e.g., exposure to terrorism and security threats) (31–34). In these cases, it may even be better to use emotion-focused coping, since this strategy may reduce the negative psychological effects of the scenario/event without confronting it directly (30). The pandemic studied here certainly fits the definition of an event perceived as uncontrollable or lacking a viable solution. It is logical for participants to use emotional (e.g., concerns about health as well as about one's financial situation) in conjunction with practical coping strategies (e.g., attempts to protect themselves as well as their family).

Finally, although at the time the study was conducted there was no indication that the coronavirus acted differently among men and women, the findings show that women had higher levels of psychological distress compared to men. This is in line with previous findings showing that women appear to be more vulnerable to internalizing symptoms, both in studies on the coronavirus (1) as well as in national studies on psychological distress levels (35, 43, 44). This tendency is well-documented and has been attributed to physiological differences (45), differences in cognitive appraisal and coping (46), socialization, and social factors (47).

To sum, due to the unusual nature of the research subject, the current study can be seen as exploratory. Psychological and coping responses following infectious disease outbreaks are relatively understudied. Thus, the findings should be approached with appropriate caution. In addition, our online survey sampling method has its befits and drawbacks. As for the former, online surveys allow for faster data collection and access to a potentially more diverse pool of participants. However, for the latter, some degree of potential sample bias should be taken into account. This strategy was not based on a random selection of the sample, and the study population did not reflect the actual pattern of the general population. In addition, there was no measurement of prepandemic of the variables we measured in the current study. Thus, It is possible that differences reflect pre-pandemic patterns.

Online surveys can reach only those who are online and those who agree to be part of the panel, and not all those who are invited to respond. In addition, our results rely on self-report questionnaires. Self-reported levels of psychological impact may not always be aligned with assessment by mental health professionals (48). Although this type of research design is generally a reliable source for gathering information about people's experiences, including regarding exposure to stressful events (1, 3), a multi-informant paradigm could enhance the data. Finally, only a single participant had contracted the virus. Thus, the findings could not be generalized to confirmed or suspected cases of COVID-19. However, in a time of crisis such as the current COVID-19 pandemic, there is a need to rapidly develop ways to better detect and classify those at greatest risk (49).

Overall, the research results indicate that although the coronavirus posed a higher psychological risk for older adults, it seems that this age group was better able to cope with its psychological effects, at least in Israel where the number of those infected was low. Qiu et al. (1) indicate that in regions where there seem to be better medical resources and control measures were taken against the pandemic, psychological distress was lower. We assume that knowledge that medical staff and resources in Israel are known to be on a high standard and about the drastic steps that were taken almost from the beginning of the coronavirus spread, resulted in the low psychological distress levels found among all age groups in the current study and especially among older adults. However, in the current study we have examined age as an objective variable. it may be that age should also be considered as a subjective perceived factor, which was found to be related to the mental health of older adults during the COVID-19 pandemic (50). Finally, the results of the study suggest the need to consider the younger age group as a risk group, and this needs to be addressed as the focus of an intervention program.

In 1919, following the influenza pandemic, Soper (51) wrote a paper that was published in *Science*, describing the feelings aroused by the flu:

“The pandemic which has just swept round the earth has been without precedent… never before has there been a catastrophe at once so sudden, so devastating and so universal. The most astonishing thing about the pandemic was the complete mystery which surrounded it… Nobody seemed to know what the disease was, where it came from or how to stop it. Anxious minds are inquiring to-day whether another wave of it will come again… Nobody can now speak authoritatively upon this subject… (p. 501)”.

Although a century has passed, the description also fits the COVID-19 crisis. Despite the relatively low rates of distress found among participants in Israel, findings from other countries (such as China) indicate extreme rates of distress and many are still in a state of uncertainty.

Any major epidemic outbreak has negative effects on individuals and on society. No country alone can prevent a global risk such as COVID-19. This shows the importance of pre-establishing community coalitions to mobilize resources efficiently and effectively and to respond successfully to the disaster-related mental health needs of affected individuals and raises the need for developing practical community mental health programs for future infectious disease outbreaks.

## Data Availability Statement

The raw data supporting the conclusions of this article will be made available by the authors, without undue reservation.

## Ethics Statement

The study was approved by the ethical standards of the Institutional Review Board (IRB) of Ariel University, Israel. The patients/participants provided their written informed consent to participate in this study.

## Author Contributions

MS and AL contributed to conception and design of the study and manuscript writing, revision, and approved the submitted version. MS led questionnaire development and organized the database.

## Conflict of Interest

The authors declare that the research was conducted in the absence of any commercial or financial relationships that could be construed as a potential conflict of interest.

## References

[1] Qiu J Shen B Zhao M Wang Z Xie B Xu Y. A nationwide survey of psychological distress among Chinese people in the COVID-19 epidemic: implications and policy recommendations. General Psychiatry. (2020) 33:e100213. 10.1136/gpsych-2020-100213 32215365 PMC7061893

[2] Itzhaky L Gelkopf M Levin Y Stein JY Solomon Z. Psychiatric reactions to continuous traumatic stress: a latent profile analysis of two Israeli samples. J Anxi Disord. (2017) 51:94–100. 10.1016/j.janxdis.2017.06.006 28709689

[3] Shechory Bitton M Laufer A. PTSD and posttraumatic growth among Israeli mothers: Opposite facets of exposure to terrorism. Stress Health. (2017) 33:676–83. 10.1002/smi.2754 28371287

[4] Levav I Novikov I Grinshpoon A Rosenblum J Ponizovsky A. Health services utilization in Jerusalem under terrorism. Am J Psychiatry. (2006) 163:1355–61. 10.1176/ajp.2006.163.8.1355 16877647

[5] Ponizovsky AM Haklai Z Goldberger N. Association between psychological distress and mortality: the case of Israel. J Epidemiol Commun Health. (2018) 72:726–32. 10.1136/jech-2017-210356 29599386

[6] Kimhi S Marciano H Eshel Y Adini B. Resilience and demographic characteristics predicting distress during the COVID-19 crisis. Soc Sci Med. (2020) 265:113389. 10.1016/j.socscimed.2020.113389 33039732 PMC7518838

[7] Palgi Y Shrira A Ring L Bodner E Avidor S Bergman Y. The loneliness pandemic: loneliness and other concomitants of depression, anxiety and their comorbidity during the COVID-19 outbreak. J Affect Disord. (2020) 275:109–111. 10.1016/j.jad.2020.06.036 32658811 PMC7330569

[8] Brooks SK Webster RK Smith LE Woodland L Wessely S Greenberg N. The psychological impact of quarantine and how to reduce it: rapid review of the evidence. Lancet. (2020) 395:912–20. 10.1016/S0140-6736(20)30460-8 32112714 PMC7158942

[9] Huang C Wang Y Xingwang L Ren L Zaho J Hu Y. Clinical features of patients infected with 2019 novel coronavirus in Wuhan, China. Lancet. (2020) 395:497–506. 10.1016/S0140-6736(20)30183-5 31986264 PMC7159299

[10] Xiong J Lipsitz O Nasri F Lui LM Gill H Phan L. (2020). Impact of COVID-19 pandemic on mental health in the general population: a systematic review. J Affect Disord. 277:55–64. 10.1016/j.jad.2020.08.001 32799105 PMC7413844

[11] Bonanno GA. Loss, trauma, and human resilience: have we underestimated the human capacity to thrive after extremely aversive events? Am Psychol. (2004) 59:20–8. 10.1037/0003-066X.59.1.20 14736317

[12] Straud C Henderson SN Vega L Black R Van Hasselt V Resiliency and posttraumatic stress symptoms in Firefighter paramedics: the mediating role of depression, anxiety, and sleep. Traumatology. (2017) 24:140–7. 10.1037/trm0000142

[13] Davydov DM Stewart R Ritchie K Chaudieu I. Resilience and mental health. Clin Psychol Rev. (2010) 30:479–95. 10.1016/j.cpr.2010.03.003 20395025

[14] Connor KM Davidson JR. Development of a new resilience scale: the Connor-Davidson Resilience Scale (CD-RISC). Depress Anxiety. (2003) 18:76–82. 10.1002/da.10113 12964174

[15] Bensimon M Elaboration on the association between trauma, PTSD and posttraumatic growth: the role of trait resilience. Pers Individ. Dif. (2012) 52:782–7. 10.1016/j.paid.2012.01.011

[16] Hu T Zhang D Wang J A meta-analysis of the trait resilience and mental health. Pers. Individ. Dif. (2015) 76:18–27. 10.1016/j.paid.2014.11.039

[17] Besser A Zeigler-Hill V Weinberg M Pincus AL Neria Y Intrapersonal resilience moderates the association between exposure-severity and PTSD symptoms among civilians exposed to the 2014 Israel–Gaza conflict. Self Identity. (2015) 14:1–15. 10.1080/15298868.2014.966143

[18] Reich JW Zautra AJ Hall JSE Handbook of Adult Resilience. New York, NY: The Guilford Press (2010).

[19] Lazarus RS Folkman S Stress Appraisal and Coping. New York, NY: Springer (1984).

[20] Lazarus RS Folkman S The concept of coping. In: Monat A Lazarus R editors. Stress and Coping. New York, NY: Columbia University Press (1991). p. 91–226.

[21] Braun-Lewensohn O Coping strategies as mediators of the relationship between chronic exposure to missile attacks and stress reactions. J Child Adolescent Trauma. (2012) 5:315–26. 10.1080/19361521.2012.719596

[22] Thompson NJ Fiorillo D Rothbaum BO Ressler KJ Michopoulos V. Coping strategies as mediators in relation to resilience and posttraumatic stress disorder. J Affect Disord. (2018) 225:153–9. 10.1016/j.jad.2017.08.049 28837948 PMC5626644

[23] Folkman S. Stress, coping, and hope. In Carr BI Steel J editors. Psychological Aspects of Cancer. New York, NY: Springer (2013). p. 119–27. 10.1007/978-1-4614-4866-2_8

[24] Gilbar O Plivzky N Gil S Counterfactual thinking, coping strategies and coping resources as predictors of PTSD diagnosed in physically injured victims of terror attacks. J Loss Trauma. (2010) 15:304–24. 10.1080/15325020903382350

[25] Taft CT Vogt DS Mechanic MB Resick P. Women seeking help for relationship aggression. J Family Psychol. (2007) 21:354–62. 10.1037/0893-3200.21.3.354 17874920 PMC2971557

[26] Li MH Nishikawa T The relationship between active coping and trait resilience across U.S. and Taiwanese college student samples. J College Counsel. (2012) 15:157–71. 10.1002/j.2161-1882.2012.00013.x

[27] Ben-Zur H Gilbar O Lev S. Coping with breast cancer: Patient, spouse and dyadic models. Psychosom Med. (2001) 63:32–9. 10.1097/00006842-200101000-00004 11211062

[28] Carver CS Scheier M Vigilant and avoidant coping in two patient groups. In: Krohne HW editor. Attention and Avoidance. Seattle, WA: Hogrefe & Huber (1993). p. 295–319.

[29] Rodrigues CS Renshaw KD. Associations of coping processes with posttraumatic stress disorder symptoms in national guard/reserve service members deployed during the OEF-OIF era. J Anxiety Disord. (2010) 24:694–9. 10.1016/j.janxdis.2010.04.013 20494548

[30] Zeidner M Individual differences in psychological reactions to terror attack. Pers Individ. Dif. (2006) 40:771–81. 10.1016/j.paid.2005.09.003

[31] Besser A Neria Y When home isn't a safe haven: Insecure attachment orientations, perceived social support, and PTSD symptoms among Israeli evacuees under missile threat. Psychol Trauma. (2012) 4:34–46. 10.1037/a0017835

[32] Braun-Lewensohn O Mosseri Rubin M Personal and communal resilience in communities exposed to missile attacks: does intensity of exposure matter? J Posit Psychol. (2014) 9:175–82. 10.1080/17439760.2013.873946

[33] May DC Herbert J Cline K Nellis A Predictors of fear and risk of terrorism in a rural state. Int J Rural Criminol. (2011) 1:1–22. 10.18061/1811/51129

[34] Shechory Bitton M Cohen Louck K. Does fear of terrorism differ from fear of crime and sexual assault: a question of geographical location and residential area. Int J Offender Ther Comp Criminol. (2016) 62:806–26. 10.1177/0306624X16658472 27383073

[35] Kessler RC Berglund P Demler O Jin R Merikangs KR Walters EE. Lifetime prevalence and age of onset distributions of DSM-IV Disorders in the national comorbidity survey replication. Arch Gen Psychiatry. (2005) 62:593–602. 10.1001/archpsyc.62.6.593 15939837

[36] Carver CS Scheier MF Weintraub K. Assessing coping strategies: a theoretically based approach. J Pers Soc Psychol. (1989) 56:267–83. 10.1037/0022-3514.56.2.267 2926629

[37] Shechory Bitton M PTSD, Post-traumatic growth, and coping among ultra-Orthodox Jewish battered women in Israel. J Loss Trauma. (2014) 19:155–72. 10.1080/15325024.2012.760383

[38] Derogatis LR Administration, Scoring, and Procedures Manual. Minneapolis, MN: NCS Pearson (2000).

[39] Derogatis LR BSI Brief Symptom Inventory. Administration, Scoring, and Procedures Manual (4th ed). Minneapolis, MN: National Computer Systems (1993).

[40] Losada-Baltar A Jiménez-Gonzalo L Gallego-Alberto L Pedroso-Chaparro MDS Fernandes-Pires J Márquez-González M. (2020). “We're staying at home”. association of self-perceptions of aging, personal and family resources and loneliness with psychological distress during the lock-down period of COVID-19. J Gerontol B Psychol Sci Soc Sci. gbaa048. 10.1093/geronb/gbaa048 32282920 PMC7184373

[41] Ni C Chow MC Jiang X Li S Pang SM. Factors associated with resilience of adult survivors five years after the 2008 Sichuan earthquake in China. PLoS ONE. (2015) 10:e0121033. 10.1371/journal.pone.0121033 25811775 PMC4374963

[42] Sim K Sim K Chan YH Chong PN Chua HC Soon SW. Psychosocial and coping responses within the community health care setting towards a national outbreak of an infectious disease. J Psychosom Res. (2010) 68:195–202. 10.1016/j.jpsychores.2009.04.004 20105703 PMC7094450

[43] Norris FJ Perilla JL Ibanez GE Murphy AD Sex differences in symptoms of posttraumatic stress: does culture play a role? J Trauma Stress. (2001) 14:7–28. 10.1023/A:1007851413867

[44] Shechory Bitton M Cohen Louck K. Spousal coping strategies in the shadow of terrorism. J Interpers Violence. (2017) 886260517744191. 10.1177/0886260517744191. [Epub ahead of print]. 29295009

[45] Olff M Sex and gender differences in post-traumatic stress disorder: an update. Eur J Psychotraumatol. (2017) 8(sup4):1351204 10.1080/20008198.2017.1351204

[46] Spindler H Elklit A Christianses DM. Risk factors for posttraumatic stress disorder following an industrial disaster in a residential area: a note on the origin of observed gender differences. Gend Med. (2010) 7:156–65. 10.1016/j.genm.2010.04.001 20435278

[47] Gavaranidou M Rosner R. The weaker sex? Gender and post-traumatic stress disorder. Depress Anxiety. (2003) 17:130–9. 10.1002/da.10103 12768647

[48] Wang C Pan R Wan X Tan Y Xu L Ho CS. Immediate psychological responses and associated factors during the initial stage of the 2019 coronavirus disease (COVID-19) epidemic among the general population in China. Int J Environ Res Public Health. (2020) 17:1729. 10.3390/ijerph17051729 32155789 PMC7084952

[49] Horesh D Brown AD. Traumatic stress in the age of COVID-19: a call to close critical gaps and adapt to new realities. Psychol Trauma. (2020) 12:331–5. 10.1037/tra0000592 32271070

[50] Shrira A Hoffman Y Bodner E Palgi Y COVID-19 Related loneliness and psychiatric symptoms among older adults: the buffering role of subjective age. Am J Geriatric Psychiatry. 28911:1200–4. 10.1016/j.jagp.2020.05.018 PMC7251397 32561276

[51] Soper G. The lessons of the pandemic. Science. (1919) 49:501–6. 10.1126/science.49.1274.501 17793800

